# Serum Trace Element Levels and Their Correlation with Picky Eating Behavior, Development, and Physical Activity in Early Childhood

**DOI:** 10.3390/nu13072295

**Published:** 2021-07-02

**Authors:** Hsun-Chin Chao, Jang-Jih Lu, Chang-Yo Yang, Pai-Jui Yeh, Shih-Ming Chu

**Affiliations:** 1 Division of Pediatric Gastroenterology, Department of Pediatrics, Chang Gung Children’s Medical Center, Chang Gung Memorial Hospital, Taoyuan City 33305, Taiwan; charlie01539@hotmail.com; 2 College of Medicine, Chang Gung University, Taoyuan City 33302, Taiwan; janglu45@gmail.com (J.-J.L.); changyo@cgmh.org.tw (C.-Y.Y.); kz6479@cgmh.org.tw (S.-M.C.); 3 Department of Laboratory Medicine, Chang Gung Memorial Hospital, Taoyuan City 33305, Taiwan; 4 Division of Neonatology, Department of Pediatrics, Chang Gung Children’s Medical Center, Chang Gung Memorial Hospital, Taoyuan City 33305, Taiwan

**Keywords:** trace element, picky eating behavior, development, physical activity, early childhood

## Abstract

Trace elements are vital components for healthy growth, development, and physical activity. The aim of this study was to investigate the relationship between trace element (iron, zinc, copper) deficiencies and picky eating behavior, development level, and physical activity level. This cross-sectional study involved 203 children aged 4–7 years; picky eating behavior, development level, and physical activity level were assessed through questionnaires. Zinc deficiency has the highest prevalence (37.4%); 67.5% of the children were assessed as picky eaters. Children with picky eating behaviors, poor development level, or poor physical activity level have significantly lower zinc levels, and higher prevalence of zinc deficiency. Pearson’s correlation coefficient indicated a positive correlation between serum zinc level and development scores (*r* = 0.221, *p* = 0.002) and physical activity scores (*r* = 0.469, *p* < 0.001). In multivariate analysis, zinc deficiency independently related to picky eating (OR = 2.124, *p* = 0.037, CI = 1.042–4.312), developmental level (OR = 0.893, *p* = 0.022, CI = 0.810–0.984), and physical activity level (OR = 0.785, *p* < 0.001, CI = 0.700–0.879). In conclusion, the prevalence of zinc deficiency in children aged 4–7 was high, especially in picky eaters. Zinc deficiency was significantly associated with low development and poor physical activity in early childhood.

## 1. Background

Micronutrient deficiency is an important nutritional problem, which is widespread in many developing and affluent countries [1,2,3]. Trace elements are essential nutrients involved in regulating the immune and antioxidant functions of the body, because trace elements can act as essential components or cofactors of enzymes in metabolism [4,5]. Trace element deficiencies are common among many developing countries, particularly among children, partly because of their higher physiological requirements and lower consumption of nutrient-rich foods [6]. In addition to the common iron deficiencies, increasing research results demonstrated that trace elements zinc and copper are also common deficiencies and are closely related to health problems, such as growth retardation and disease [7,8,9,10]. Iron deficiency has been associated with delayed cognitive and motor development, and iron deficiency anemia adversely affects cognitive function and psychomotor development [11]. Zinc deficiency may affect cognitive development by causing changes in neuropsychological behavior, and motor development [12]. Copper deficiency may cause both hematological and neurological disease [13].

The rapid growth of preschool children increases the need for nutrition, and malnutrition of this period may have long-term developmental consequences, such as delayed development, psychomotor delay and behavioral disorders [14,15]. Unhealthy eating habits, such as children’s picky eating or caregivers’ feeding practice, may lead to excessive intake of certain foods and unbalanced diets, which may result in obesity, overweight and insufficient intake of trace elements [16,17,18,19,20]. The characteristics of picky eating behaviors usually include food refusal, food neophobia, and other abnormal eating behaviors [21]. Several studies demonstrated that children with picky eating habits are associated with lower height, underweight and lower BMI [22,23], and tend to show lower attention spans, poor interpersonal relationships, and low stair-climbing activity [24,25]. 

Limited data regarding the prevalence of trace element deficiency in Taiwanese children, from a study using big data analysis of disease incidence, found that poverty was significantly associated with iron deficiency anemia in elementary school-aged children [26], and a recent longitudinal study observed a high prevalence of iron deficiency at 1 year but low at 3 years and zinc deficiency was uncommon before 3 years old [27]. Little is known about the association of trace element deficiencies with picky eating behaviors, development state, and physical activity level, especially for children in the developmental stage. We hypothesize that trace element (iron, zinc, and copper) deficiencies may contribute to poor development status and low physical activity levels based on their physiological role, and young children with picky eating behaviors had high prevalence of trace element deficiencies due to inadequate or unbalanced diets. Therefore, the study aims to investigate the prevalence of iron, zinc, and copper deficiencies among young children in Taiwan, and their relationship with picky eating behaviors, development, and physical activity.

## 2. Materials and Methods

### 2.1. Study Design and Participants

A cross-sectional descriptive study with blood examinations of serum trace element (zinc, iron copper) and structured questionnaires to assess outcome predictors (picky eating behaviors, development, and physical activity) were conducted in children aged 4–7 years. The associations between trace elements and outcome were evaluated.

This study was conducted in accordance with the Declaration of Helsinki, and this study protocol was approved by the Institutional Review Board of the Human Research Committee of Chang Gung Memorial Hospital (Approval number: 201801719AC601). Parents or guardians of eligible participants provided informed written consent. Potential participants were recruited from a well-baby or nutrition counseling clinic in Chang Gung Children’s medical center in Chang Gung Memorial Hospital. The participants received the initial screen for their eligibility. The exclusion criteria are children with chronic illnesses, whose diseases may affect eating habits, physical activity, or nutritional status, such as dental diseases, anemia, organic diseases, mental disorders, genetic diseases and psychiatric illness. If the family’s financial capacity cannot adequately support their child’s diet and nutrition, or the parents/caregivers had an inadequate concept of a child’s nutritional support, development and physical activities, these potential participants were also excluded. Besides, the targeted participants were accordingly selected for the concern of the precision of the questionnaires being filled out. We observed that less-privileged caregivers would have less resources and time to monitor their children. Therefore, we screened for caregivers who have ample opportunities to observe and accompany their children.

**Figure 1:**
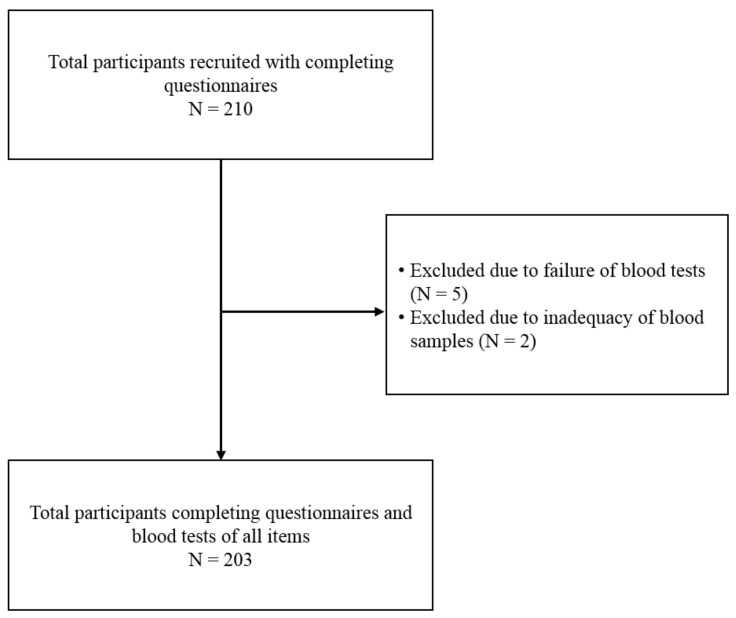
Flow chart of patient selection for the study.

From January 2019 to December 2020, a total of 210 children (106 boys and 104 girls) were willing to participate in this study. Due to the refusal to draw blood (5 children) and insufficient blood samples to complete blood analysis (2 children), 7 children withdrew from this study. Finally, a total of 203 children were enrolled in this study and completed the questionnaire and blood test. Figure 1 shows the flow chart of study population selection.

The interviewers employed were those who had a good education level (college or higher), and qualified interviewing skills including clarification of the questionnaires, unbiased skills of being neutral to the respondents, avoidance of using their own definitions to any phrase, attempt of encouragement in answers that were more complete, and thoughtful, and providing their respondents with appropriate feedback. 

Face-to-face interviews were conducted with parents/caregivers of all participants for about 30 min to collect sociodemographic data, including the age, gender, weight and height and body mass index (BMI) (weight (kg)/height (m^2^)) of the children. Picky eating behaviors and dietary habits were assessed by a food and eating questionnaire, development and physical activity assessed by a general questionnaire. The food, general health and medical questionnaires comprised closed-ended categorical questions, including children’s food preferences, dietary habits, parent/child interactions during meal times, language ability, developmental behaviors, levels of physical activities, and medical records in the last year. Before the assessment, each caregiver would receive instruction on how to fill out the questionnaires, and we also expressed the importance of cooperating with the opinions from the kindergarten (or school) teacher or other close observers during the interview. 

The primary endpoint of this study was the prevalence of trace element (iron, zinc, copper) deficiencies and the correlations between serum levels of trace elements (iron, zinc, copper) and picky eating behaviors, development status, and physical activity levels. The secondary endpoint was comparing the relationship between trace element (iron, zinc, copper) deficiencies and picky eating behaviors, lower development status, or lower levels of physical activity, and measuring the strength of correlation for these categorical parameters to provide an understanding of the strength and direction of the relationship between the two variables. 

### 2.2. Assessment of Picky Eating Behaviors of the Children

The picky eating behaviors of the children were evaluated by the questionnaire [25], which included a structured questionnaire to assess the food preferences of the children and a separate section of the questionnaire to assess parental feeding behavior (6 items: 4 appropriate behaviors and 2 inappropriate behaviors) and children’s eating behaviors (6 items: 2 healthy eating behaviors and 4 picky eating behaviors). The structured questionnaire for children’s food preferences is based on the United Kingdom Department of Health Survey of the Diets of British School Children [28] and the dietary assessment among school-aged children [29] as references, and was then further adjusted according to the dietary culture and food habits of Taiwanese. 

The questionnaire of food preferences included 2 major items: (1) child’s foods (meals) and their preferences in 7 food categories: (i) grains (rice, bread, cereals, potato, noodles, etc.); (ii) protein foods (meats, fish, seafood, beans, etc.); (iii) vegetables; (iv) fruits; (v) dairy foods (milk, cheese, yogurt, etc.); (vi) fats and oils (vegetable oil, butter, cream, salad, etc.); and (vii) snacks and sweets (candy, cookie, cake, etc.) in the past 2 weeks; (2) preferences in familiar foods (list of 40 foods for regular meals). The answers were “tried and not tried” in each food and responding to preferences of the tried foods. Items were scored on a 5-point scale as “like very much”, “like moderately”, “neither like or dislike”, “dislike moderately”, and “dislike very much”. The eating behavior questionnaires were based on the study of Wardle et al. on the trends of eating behaviors in preschool children [30]. The 4 questions of the picky eating behavior questionnaire included: (i) eating limited foods (usually eating fixed foods or having a strong preference for specific foods, such as cooked foods, milk, or sweets); (ii) unwillingness to eat regular meals; (iii) unwillingness to try new foods; and (iv) refusal of one or multiple food groups in 6 major food groups (grains, protein foods, vegetables, fruits, dairy foods, and fats and oil). The scale is scored as follows: never = 1; rarely = 2; sometimes = 3; often = 4; always = 5. Mean scores were calculated for each subscale (range 1–5) with higher scores indicating higher values of each trait. In this study, picky eating is defined as a positive response of “always” to at least one item of the picky eating behaviors on questionnaire of eating behaviors based on the studies of Jacobi et al. and Toyama et al. [31,32].

### 2.3. Assessment of Development of the Children

The questionnaires used to assess child development were based on the Bayley-IV scale for infant and toddlers and interdisciplinary assessment for children development conducted by the Ministry of Health and Welfare of R.O.C. [33]. The content of the questionnaire included 7 items in 3 categories: learning ability (2 items: attention and learning), verbal development (2 items: verbal development, language learning, confluence in speech), and interpersonal relationships (3 items: adaptation to new environments, cooperation, adaptation of being separated from relatives). Respondents were asked to rate the level of agreement with the question on a five-point scale ((unacceptable (score = 1), improvement expected (score = 2, acceptable (score = 3), exceeding expected (score = 4), outstanding (score = 5)). Mean scores were calculated for each subscale (range 1–5) with higher scores indicating higher values of each trait. Poor development status is defined as the total score ≤ 21 (total score range 7–35). 

### 2.4. Assessment of Physical Activity of the Children

The questionnaire for assessing physical activity was based on the studies of Reilly et al. and Lobelo et al. [34,35]. The content of the questionnaire includes four items: normal-pace walking, sport activities, stair-climbing and running. Respondents were asked to rate the level of agreement with the question on a five-point scale ((unacceptable (score = 1), improvement expected (score = 2, acceptable (score = 3), exceeding expected (score = 4), outstanding (score = 5)). Mean scores were calculated for each subscale (range 1–5) with higher scores indicating higher values of each trait. A child with a total score ≤ 12 is defined as having a poor physical activity level (total score range 4–20). 

### 2.5. Measurement of Serum Trace Element Levels in Children

The participant was invited for blood sampling in a nearby phlebotomy site established for the survey. A non-fasting peripheral venipuncture blood sample was taken from each child in the sitting position. One blood sample was drawn into a trace element (TE)-free evacuated tube (Beckton Dickinson, Franklin Lakes, NJ), and a second into an evacuated tube containing EDTA as an anticoagulant (Becton Dickinson, Franklin Lakes, NJ). All blood samples were refrigerated immediately after collection [36], and the serum from the TE-free tubes separated within two hours using TE-free techniques [37] and protected from ultraviolet light. The dried blood spots were protected from light and stored in a plastic bag with desiccant, and the packaged dried blood spots were preserved in a refrigerator (2–8 °C). Blood tests were performed to examine the cell blood count (CBC) and serum level of ferritin, iron, zinc, and copper. Automatic cell counter was used to determine the red blood cell count and hemoglobin level. Chemiluminescence immunoassay was used to determine serum concentrations of ferritin. Colorimetric method was used to determine the serum concentrations of iron. Atomic absorption spectroscopy was used to determine the serum concentration of zinc and copper. Iron deficiency anemia (IDA) consisted of coexistent anemia and iron deficiency. According to the World Health Organization (WHO) criteria, anemia in children aged 6 months to 5 years is defined as hemoglobin lower than 11 g/dL [38]. Severe anemia, moderate anemia and mild anemia are defined as hemoglobin concentration < 7 g/dL, 7–9.9 g/dL and 10–10.9 g/dL, respectively [39]. Iron deficiency is defined as plasma ferritin < 12 μg/L [40]. Zinc deficiency is defined as serum zinc concentration < 10.7 μmol/L (69.8 μg/dL), and copper deficiency is defined as serum copper < 90 ug/dL (14.2 pmol/L) [41,42,43,44].

### 2.6. Statistical Analyses

The correlations between trace element (serum levels, and deficiency) and outcomes (picky eating behavior, development status, and physical activity level) were evaluated. Statistical analyses were performed using SPSS Statistics version 20 (SPSS Inc., Chicago, IL, USA). The differences between groups were compared using Student’s *t*-test for numeric parameters and Chi-square test for non-numeric parameters. Phi correlation coefficient was performed to measure the strength of correlation for categorical parameters. Pearson’s correlation coefficient was used to analyze the association between the numerical parameters. The level of significance was set at *p*-value less than 0.05. Logistic regression was used to determine the association of each trace element deficiency with factors of demographics, picky eating behaviors, development status, and physical activity level. The potential predictors (age, gender, weight, height, BMI) considered for inclusion in the models were measures of demographics and clinical factors influencing trace element deficiency in the physiologic framework. The multivariate analysis combines the significant factors of univariate analysis and through multivariate analysis to determine the independent predictors of outcomes. Odds ratio (OR) and 95 % confidence intervals (CI) were estimated using univariate and multivariate binary logistic regression analysis. All statistical analyses were two-tailed and considered significant at *p* < 0.05. 

## 3. Results

### 3.1. Demographic and Clinical Characteristics

The demographic characteristics of the participants are shown in Table 1. A total of 203 participants completed the questionnaire and blood tests, including 102 boys (50.2%) and 101 girls (49.8%), with a mean age of 5.21 ± 0.85 years. The mean body height, weight, and BMI were 107.90 ± 7.51 cm, 18.22 ± 3.85 kg, and 15.49 ± 2.12, respectively. After the food and dietary questionnaire survey, 137 children (67.5 %) were found to have picky eating behavior. The mean development score and physical activity score of these children were 22.41 ± 3.57, and 15.81 ± 3.16, respectively. According to the assessment of the children’s development level, 102 children (50.2%) have good developmental levels (total score > 21). Most children (159; 78.3%) have good physical activity levels (total score > 12).

Table 2 shows the children’s biochemical data. The mean hemoglobin, ferritin, and serum iron levels of these children were 12.75 ± 0.78 g/dL, 45.86 ± 25.79 ng/mL, and 82.37 ± 32.10 μg/dL, respectively. There were 33 children (16.3%) with low iron levels (iron deficiency), but none of them have iron-deficiency anemia (IDA). The mean serum zinc level was 74.46 ± 10.91 μg/dL, while the mean copper levels were 114.92 ± 22.96 μg/dL. A total of 20 children (9.9%) had low copper levels (copper deficiency). Compared with iron and copper deficiencies, these children have a higher prevalence of zinc deficiency (76 children; 37.4%). A total of 100 children (49.3%) had single trace element deficiency (iron, zinc or copper), and 28 children (13.8%) had multiple trace element deficiencies. Among these 28 children, 18 children (64.3%) had both iron and zinc deficiencies, while none of them had iron, zinc and copper deficiencies at the same time. 

### 3.2. Correlation of Trace Element Levels and Deficiencies with Picky Eating Behaviors Development and Physical Activity

Table 3 shows the correlation between serum iron, zinc and copper trace element levels and the picky eating behaviors of children. The results showed that the serum zinc level of children with picky eating behaviors were significantly lower than those of children without picky eating behaviors (73.35 ± 11.97 vs. 76.67 ± 8.10 μg/dL, *p* = 0.004). In addition, compared with non-picky eaters, picky eaters also have lower serum iron levels (81.53 ± 32.42 μg/dL) and higher serum copper levels (115.74 ± 23.45 μg/dL). In addition to being associated with lower trace element levels, picky eating behavior is also associated with higher prevalence of trace element deficiencies. Children with picky eating behaviors have a significantly higher prevalence of zinc deficiency than children without picky eating behaviors (43.1% vs. 25.8%, *p* = 0.026). In addition, the prevalence of iron deficiency (16.8% vs. 15.2%) and copper deficiency (10.9% vs. 7.6%) in picky eaters is higher than that in non-picky eaters, although it has not yet reached statistical significance. In Phi correlation coefficient analysis, picky eating behaviors remained independently related to zinc deficiency (*p* = 0.017).

Table 4 shows the statistical analysis of the correlation between serum trace element levels and child development. Compared with children with good development levels, children with poor development levels have significantly lower serum zinc levels (76.14 ± 10.67 vs. 72.73 ± 10.19 μg/dL, *p* = 0.042). Children with poor development levels have a significantly higher prevalence of zinc deficiency than children with good development levels (46% vs. 30.7%, *p* = 0.036). Although the results did not reach statistical significance, children with poor development levels had a higher prevalence of iron (16.9% vs. 15.8%), zinc (40.6% vs. 30.7%) and copper (10.1% vs. 9.6%) deficiencies. In Phi correlation coefficient analysis, developmental levels remained independently related to zinc deficiency (*p* = 0.025).

Table 5 shows the statistical analysis of the correlation between serum trace element levels and children’s physical activity levels. Compared with children with good physical activity levels, children with poor physical activity levels have significantly lower serum zinc levels (76.41 ± 10.93 vs. 67.38 ± 8.21 μg/dL, *p* = 0.008) and a higher prevalence of zinc deficiency (63.6% vs. 30.2%, *p* < 0.001). In addition, although the statistical results were not significant, children with poor physical activity have lower serum iron levels (82.71 ± 32.61 vs. 81.93 ± 31.62 μg/dL) and have a higher prevalence of iron deficiency (15.7% vs. 18.2%). In Phi correlation coefficient analysis, physical activity levels remained independently related to zinc deficiency (*p* < 0.001).

Table 6 shows the association of trace element deficiencies and the development and physical activity of children. Consistent with the above results, children with zinc deficiency had significantly lower development scores (21.25 ± 3.88 vs. 23.09 ± 3.15, *p* = 0.005) and physical activity scores (14.26 ± 2.99 vs. 16.75 ± 2.43, *p* < 0.001) than those without zinc deficiency (*p* = 0.005, and <0.001). 

Table 7 shows the Pearson’s correlation coefficient between trace element levels and the scores of development and physical activity. Pearson correlation coefficient indicated a positive correlation between serum zinc level and development scores (*r* = 0.221, *p* = 0.002) and physical activity scores (*r* = 0.469, *p* < 0.001). The test did not show a correlation between the other trace elements (iron, copper) and scores of development and physical activity (Table 7). 

### 3.3. Correlation of Trace Element Deficiencies with Demographics and Clinical Characteristics

Logistic regression analysis was further conducted to assess the association between trace element deficiencies and factors of demographics, picky eating, development and physical activity (Table 8, Table 9 and Table 10). As shown in Table 8, zinc deficiency was not associated with children age, gender, weight, height, and BMI (*p* > 0.05). However, zinc deficiency was significantly associated with picky eating behavior (OR = 2.180), development level (OR = 0.852), and physical activity level (OR = 0.755). In multivariable analysis, zinc deficiency remained independently related to: picky eating (OR = 2.124, *p* = 0.037, CI = 1.042–4.312), developmental level (OR = 0.893, *p* = 0.022, CI = 0.810–0.984), and physical activity level (OR = 0.785, *p* < 0.001, CI = 0.700–0.879) (Table 8). The test did not show correlation between the other trace element (iron, copper) deficiency and picky eating behavior, development level, and physical activity level (*p* > 0.05, Table 9 and Table 10), whereas copper deficiency was significantly associated with demographic factors: age (OR = 0.526), weight (OR = 0.779), and height (OR = 0.914) (Table 10). In multivariable analysis, weight was the only independent factor associated with copper deficiency (OR = 0.779, *p* = 0.008, CI = 0.649–0.936). 

## 4. Discussion

Although several studies have investigated the prevalence of trace element deficiencies among children [45,46,47,48], few have explored the correlation between trace element deficiencies and children’s picky eating behaviors, development, and physical activity. This study aimed to evaluate the levels of iron, zinc, and copper serum trace elements in children aged 4–7 in Taiwan, and to explore the correlation of trace element deficiencies and children’s picky eating behaviors, development and physical activity level. The results of the study found that even if the family’s financial capacity can adequately support the children’s diet, the prevalence of trace element deficiencies children aged 4–7 in Taiwan is still high. Nearly half of the children (49.3%) had at least one trace element deficiency, and the prevalence of zinc deficiency (37.4%) is much higher than that of iron (16.3%) and copper (9.9%) deficiency. In addition, there were 28 children suffering from multiple trace element deficiencies, of which the proportion of children with zinc and iron co-deficiency is the highest (64.3%, 18 of 28 children). Furthermore, children with zinc deficiency were significantly associated with picky eating behaviors, lower development levels and poor physical activity.

Child’s picky eating was defined variably; picky eating has been described as the consumption of an inadequate variety of foods. The picky eating defined in our study was based on the questionnaires of detecting picky eating by strong existence of picky eating behaviors picky eating behaviors on 4 questions based on the study by Wardle et al. on the trends of eating behaviors in preschool children. Picky eating behaviors included rejection of certain types of food, acceptance of only certain foods, unwillingness to try new foods (food phobia), limited intake of some food groups and strong food preferences [31]. Toyama et al. indicated that significant parent-reported feeding questions may identify persistent picky eaters at an early age [32]. The questionnaires used for picky eating in our study recruited these typical picky eating behaviors help to identify picky eaters more precisely. Picky eating behaviors are common in infancy and childhood [49]. In a large-scale survey of 7057 children aged 2–7 in Hong Kong, 43% of children were reported by their parents as picky eaters [50]. A study of 937 Chinese children aged 3–7 identified 54% as picky eaters [51]. In our previous studies, we found that 54% and 62% of Taiwanese children aged 2–4 and aged 1–10 are picky eaters, respectively [24,25]. In this study, we found that 67.5% of children aged 4–7 were picky eaters, which is slightly higher than previous studies of Asian children. This seems to indicate that the prevalence of picky eaters increases with age, which is supported by some studies [52,53,54]. 

Since the purpose of this study was to investigate the prevalence of trace element deficiencies in early school-age children who appear to be healthy, participants with iron deficiency anemia were excluded. This is because iron deficiency anemia can impair immunity and nervous system function, increase fatigue, and reduce work and exercise performance [55,56]. Even so, we still found that 16.3% of children had serum iron levels below the standard level. In addition, the results of the study showed that serum iron level may be positively correlated with the children’s development and physical activity. Children with iron deficiency had lower total scores for development and physical activity (Table 6). The statistical insignificance may be due to the small number of children with iron deficiency (n = 33). It is well known that iron deficiency may affect the development of infants and children. The study by Patricia et al. found that growth and anemia-iron status are two significant predictors of walking [57]. Iron supplementation can improve mental development scores modestly, especially for intelligence tests above 7 years of age in initially anemic or iron-deficient anemic children [58]. Physical activity is related to energy expenditure, thus the plasma mineral levels of healthy individuals also play an important role in the level of physical activity [59]. In addition to iron, physical activity and an adequate balance in zinc and copper levels play a positive role in improving depressive score among school children [60]. 

In this sample of Taiwanese healthy children aged 4–7, the proportion of children with zinc deficiency (37.4%) was much higher than that of iron and cooper deficiencies. Zinc is necessary for the structure and activity of more than 300 enzymes in various organs in the human body, and plays important roles in immune function, growth, and development [61]. In general, zinc deficiency is caused by malabsorptive diseases, insufficient intake and, to some extent, increased losses. Red meat, shellfish and oysters are good source of zinc, while phytate and fiber inhibit the absorption of zinc. Therefore, children with picky eating behaviors or consuming a primarily plant-based diet and/or cereal-based diets (high in phytates) are susceptible to zinc deficiency [15]. In this study, children with Crohn’s disease, intestinal bypass, short-bowel syndrome, and celiac disease were excluded from this study, indicating that these children with zinc deficiency are not caused by malabsorptive diseases. In addition, these children have no symptoms of acute or chronic diarrheal diseases, suggesting that diet may be the cause of the high incidence of zinc deficiency. The results of this study support this assumption that zinc deficiency was significantly associated with picky eating behavior (univariate analysis: OR = 2.180, *p* = 0.018; multivariate analysis: OR 2.124, *p* = 0.022, Table 8). The potential reasons for zinc deficiency are due to a risk of zinc deficiency in early childhood with the need of zinc for their growth, development, physical activity, and vigorous activity like running, and can occur during caloric restriction and when dietary food variety is limited. My previous research has observed that fear of unfamiliar places, and poor physical activity were significantly higher in preschool picky eaters (aged 2–4 years) [25]. In this study, we found that older children (aged 4–7 years) with zinc deficiency were significantly associated with poor development and poor physical activity. It is essential to further explore the relationship between picky eating behavior and development/physical activity levels among these children.

Poor zinc levels may result in decreased heart and lung function, as well as reduced strength and endurance. The results of this study also supported this opinion that zinc deficiency is significantly associated with children’s poor development and low physical activity (Table 6). In addition, although the impact of picky eating on the growth and weight status of children is still controversial [22,23,54,62], this study found that children with picky eating behaviors had lower zinc levels and were significantly associated with a high prevalence of zinc deficiency. In addition to improving immunity [63,64] and reducing the duration and severity of diarrhea in children [65,66], recent clinical trials have shown that zinc supplementation can improve children’s development and physical activity. A randomized clinical trial of 251 infants by Colombo et al. showed that zinc supplementation has a positive effect on of sensorimotor development [67]. A recent randomized, multicenter study by Abdollahi et al. also found that zinc supplementation can significantly increase the body length of children and prevent growth retardation [68]. Another randomized controlled trial in Thailand also indicated that children who received zinc and multivitamins had significantly higher gain in height, and the extra gain in height manifested after 2 months of supplementation [69]. In this study, children with poor development were significantly associated with lower serum zinc levels (*p* = 0.042), and logistic regression analysis displayed zinc deficiency was significantly associated with development levels (univariate: OR = 0.852, *p* < 0.001, multivariate: OR 0.893, *p* = 0.037, Table 8). To the best of our knowledge, there is still a lack of clinical studies exploring the effect of zinc supplementation in the improvement of physical activity. A study of voluntary physical activity in rats by Scrimgeour et al. found that rats fed a low-zinc diet had fewer running miles per day than rats fed a high-zinc diet [70]. In this study, children with poor physical activity were significantly associated with lower serum zinc levels (*p* = 0.008), and logistic regression analysis displayed zinc deficiency was significantly associated with physical activity levels (univariate analysis: OR = 0.755, *p* < 0.001; multivariate analysis: OR 0.785, *p* < 0.001, Table 8). Further prospective studies should be conducted, preferably a randomized controlled design, to investigate whether zinc supplementation can improve children’s physical activity and reduce the high prevalence of zinc deficiency among children in Taiwan.

The strengths of this study include its prospective design for children of specific ages and the use of extensive questionnaire to assess picky eating behaviors, developmental levels, and physical activity levels. To minimize the selection bias, the recruited participants were physically healthy, and their families do not have the economic burden of providing children with nutritional support. To strengthen the scientific credibility, the picky eating behaviors, development, and physical activity were assessed through questionnaires, and the development and physical activity of the children were scored to demonstrate the difference in development and physical activities between picky eaters and non-picky eaters. The present study has several limitations. First, the self-rating questionnaires are based on the point of view of the caregivers, and it may not fully represent the actual situation of the child. Second, since this study was a cross-sectional design, it cannot elucidate the temporal relationship between trace element deficiency, picky eating, development, and physical activity (causal inference). Third, the small numbers of children in certain subgroups, particularly those with iron deficiency, may have limited the study’s power to detect significant associations. Fourth, the groups are very different in composition (picky eaters (137) vs. non-picky eaters (66); poor development levels (89) vs. good development levels (114); poor physical activity (44) levels vs. good physical activity levels (159). Finally, since these participants in this study were recruited from northern Taiwan, the results may not fully reflect the situation of children in the other parts in Taiwan or elsewhere. We expect that a prospective, population-based study can be conducted to cover the children participants with different socio-economic status nationally in the future. 

## 5. Conclusions

The prevalence of trace element deficiency (iron, zinc, and copper) in Taiwanese children aged 4–7 years is high, especially among children who are picky eaters. Among them, the prevalence of zinc deficiency in children is highest, and serum zinc levels are significantly positively correlated with development and physically activity. In the future, it is essential to conduct a longitudinal study to evaluate the effect of oral zinc supplementation on picky eating behaviors, and the performance in development and physical activity in early childhood, which must recruit more participants from different cities and consider potential confounding factors like dietary intake and food variety that could influence an association. The application of food-frequency questionnaires and 3-day dietary records to assess intakes of energy and nutrients is anticipated.

## Figures and Tables

**Table 1:** Demographics and clinical characteristics of the subjects.

Parameters	Values
Number	203
Age (years, mean ± SD)	5.21 ± 0.85
Male (%)	102 (50.2%)
Body height	107.9 ± 7.51 cm
Body weight	18.22 ± 3.85 kg
BMI	15.49 ± 2.12
Picky eating	137 (67.5%)
Development level (score: 7–35)	22.41 ± 3.57
good (score > 21)	102 (50.2%)
poor (score ≤ 21)	101 (49.8%)
Physical activity level (score: 4–20)	15.81 ± 3.16
good (score > 12)	159 (78.3%)
poor (score ≤ 12)	44 (21.7%)

Abbreviation: SD, standard deviation. Development level: total scores were calculated on the questionnaire assessing development, which consisted of 7 items (score of 1–5 in each item). Physical activity level: total scores were calculated on the questionnaire assessing physical activity, which consisted of 4 items (score of 1–5 in each item).

**Table 2:** Laboratory data and deficiency of trace elements of the subjects.

Parameters	Values
Hemoglobin (mean ± SD)	12.75 ± 0.78 g/dL
Ferritin (mean ± SD)	45.86 ± 25.79 ng/mL
Iron (mean ± SD)	82.37 ± 32.10 ug/dL
Deficiency (<50 ug/dL)	33 (16.3%)
Zinc (mean ± SD)	74.46 ± 10.91 kg
Deficiency (<70 ug/dL)	76 (37.4%)
Copper (mean ± SD)	114.92 ± 22.96
Deficiency (<90 ug/dL)	20 (9.9%)
Trace element deficiency	
Single (iron, zinc, or copper)	100 (49.3 %)
Multiple	28 (13.8 %)
(1) Iron & zinc & copper	0 (0.0%)
(2) Iron & zinc	18 (8.9 %)
(3) Zinc & copper	8 (3.9 %)
(4) Iron & copper	2 (1.0%)

Abbreviation: SD, standard deviation.

**Table 3:** Correlation of trace element levels and deficiency with picky eating behaviors.

Variables/Groups	Picky Eating Behaviors		Student’s *t* (95% CI) ^a^/Chi-Square ^b^	*p*-Value	Phi	*p*-Value
	Yes (n = 137)	No (n = 66)				
Iron						
Level (mean ± SD)	81.53 ± 32.42	84.12 ± 31.57	−9.183, 4.003 ^a^	0.439		
Deficiency (n, %)	23 (16.8%)	10 (15.2%)	0.009 ^b^	0.926	0.021	0.767
Zinc						
Level (mean ± SD)	73.35 ± 11.97	76.67 ± 8.1	−5.549, −1.091 ^a^	0.004		
Deficiency (n, %)	59 (43.1%)	17 (25.8%)	4.982 ^b^	0.026	0.168	0.017
Copper						
Level (mean ± SD)	115.64 ± 23.45	113.41 ± 22.01	−2.485, 6.945 ^a^	0.352		
Deficiency (n, %)	15 (10.9%)	5 (7.6%)	0.254 ^b^	0.614	0.053	0.450

Abbreviation: CI, confidence interval. ^a^ Data of continuous variables were expressed as mean ± SD, and analyzed by Student’s *t*-test. ^b^ Descriptive data were analyzed by Chi-square test.

**Table 4:** Correlation of trace element levels and deficiency with development levels.

Variables/Groups	Development Levels		Student’s *t* (95% CI) ^a^/Chi-Square ^b^	*p*-Value	Phi	*p*-Value
	Poor (n = 89)	Good (n = 114)				
Iron						
Level (mean ± SD)	81.29 ± 31.53	83.21 ± 33.41	−12.21, 8.365 ^a^	0.713		
Deficiency (n, %)	15 (16.9%)	18 (15.8%)	<0.001 ^b^	0.990	0.014	0.838
Zinc						
Level (mean ± SD)	72.73 ± 10.19	76.14 ± 10.67	−6.702, −0.1182 ^a^	0.042		
Deficiency (n, %)	41 (46%)	35 (30.7%)	4.404	0.036	0.158	0.025
Copper						
Level (mean ± SD)	115.43 ± 22.13	114.45 ± 22.91	−6.145, 8.105 ^a^	0.787		
Deficiency (n, %)	9 (10.1%)	11(9.6%)	<0.001	1.000	0.008	0.912

Abbreviation: CI, confidence interval. ^a^ Data of continuous variables were expressed as mean ± SD, and analyzed by Student’s *t*-test. ^b^ Descriptive data were analyzed by Chi-square test.

**Table 5:** Correlation of trace element levels and deficiency with physical activity levels.

Variables/Groups	Physical Activity Levels		Student’s *t* (95% CI)^a^/Chi-Square ^b^	*p*-Value	Phi	*p*-Value
	Poor (n = 44)	Good (n = 159)				
Iron						
Level (mean ± SD)	81.93 ± 31.62	82.71 ± 32.61	−11.47, 29.91 ^a^	0.942		
Deficiency (n, %)	8 (18.2%)	25 (15.7%)	0.026	0.873	0.027	0.696
Zinc						
Level (mean ± SD)	67.38 ± 8.21	76.41 ± 10.93	−15.68, −2.384 ^a^	0.008		
Deficiency (n, %)	28. (63.6%)	48 (30.2%)	15.064 ^b^	<0.001	0.285	<0.001
Copper						
Level (mean ± SD)	115.36 ± 22.02	114.79 ± 23.21	−14.09, 15.23 ^a^	0.939		
Deficiency (n, %)	4 (9.1%)	16 (10.1%)	<0.001 ^b^	1.000	−0.013	0.848

Abbreviation: CI, confidence interval. ^a^ Data of continuous variables were expressed as mean ± SD, and analyzed by Student’s *t*-test. ^b^ Descriptive data were analyzed by Chi-square test.

**Table 6:** Association of trace element deficiency with development and physical activity scores.

Variables	Trace Element Deficiency		Student-*t* (95% CI)	*p*-Value
	Yes	No		
Development scores				
Iron	20.88 ± 4.41	22.72 ± 3.27	−4.791, 1.111	0.220
Zinc	21.25 ± 3.88	23.09 ± 3.15	−3.111, 0.569	0.005
Cooper	22.75 ± 4.55	22.32 ± 3.63	−4.789, 5.669	0.868
Physical activity scores				
Iron	14.82 ± 3.11	16.01 ± 3.15	−3.867, 1.483	0.381
Zinc	14.26 ± 2.99	16.75 ± 2.43	−3.471, 1.509	<0.001
Cooper	15.90 ± 3.34	15.80 ± 3.15	−5.047, 5.227	0.965

Abbreviation: CI, confidence interval. Total scores were calculated on the questionnaire assessing development, which consisted of 7 items (score of 1–5 in each item) and the questionnaire assessing physical activity, which consisted of 4 items (score of 1–5 in each item). Data of continuous variables were expressed as mean ± SD, and analyzed by Student’s *t*-test.

**Table 7:** Correlation of trace element levels with development/physical activity scores.

Parameters	Development (Score)		Physical Activity (Score)	
	r	*p*-Value	r	*p*-Value
Iron level	0.066	0.346	0.036	0.614
Zinc level	0.211	0.002	0.469	<0.001
Copper level	−0.119	0.090	−0.092	0.189

r (Pearson’s correlation coefficient).

**Table 8:** Association of zinc deficiency with demographics and clinical factors.

Variables	Univariable Analysis			Multivariable Analysis		
	OR	95% CI	*p*-Value	OR	95% CI	*p*-Value
Age	0.961	0.690–1.337	0.812			
Gender	1.165	0.659–2.058	0.599			
Weight	0.967	0.896–1.043	0.385			
Height	0.992	0.995–1.031	0.684			
BMI	0.943	0.821–1.083	0.404			
Picky Eating	2.180	1.142–4.164	0.018	2.124	1.042–4.312	0.037
Development scores	0.852	0.779–0.933	<0.001	0.893	0.810–0.984	0.022
Physical activity scores	0.755	0.677–0.842	<0.001	0.785	0.700–0.879	<0.001

Abbreviation: CI, confidence interval; OR, odd ratio. The values of beta-coefficient and 95% CI were obtained from logistic regression model.

**Table 9:** Association of iron deficiency with demographics and clinical factors.

Variables	OR	95% CI	*p*-Value
Age	0.921	0.595–1.426	0.712
Gender	0.919	0.436–1.938	0.825
Weight	0.968	0.873–1.073	0.531
Height	1.015	0.966–1.067	0.554
BMI	0.877	0.716–1.074	0.206
Picky eating	0.885	0.394–1.986	0.767
Development scores	0.987	0.888–1.096	0.802
Physical activity scores	0.961	0.856–1.080	0.503

Abbreviation: CI, confidence interval; OR, odd ratio. The values of beta-coefficient and 95% CI were obtained from logistic regression model.

**Table 10:** Association of copper deficiency with demographics and clinical factors.

Variables	Univariable Analysis			Multivariable Analysis		
	OR	95% CI	*p*-Value	OR	95% CI	*p*-Value
Age	0.526	0.283–0.976	0.042			
Gender	0.499	0.190–1.307	0.157			
Weight	0.779	0.649–0.936	0.008	0.779	0.649–0.936	0.008
Height	0.914	0.851–0.981	0.013			
BMI	0.806	0.609–1.066	0.130			
Picky eating	0.667	0.231–1.920	0.452			
Developmental scores	1.030	0.904–1.175	0.655			
Physical activity scores	0.997	0.862–1.154	0.973			

Abbreviation: CI, confidence interval; OR, odd ratio. The values of beta-coefficient and 95% CI were obtained from logistic regression model.

## Abbreviations

BMI	Body Mass Index
SD	Standard Deviation
CI	Confidence Interval
